# RANK Ligand Helps Immunity to *Leishmania major* by Skewing M2-Like Into M1 Macrophages

**DOI:** 10.3389/fimmu.2020.00886

**Published:** 2020-05-12

**Authors:** Thaís S. Rigoni, Natália S. Vellozo, Mariela Cabral-Piccin, Laryssa Fabiano-Coelho, Ulisses G. Lopes, Alessandra A. Filardy, George A. DosReis, Marcela F. Lopes

**Affiliations:** ^1^Instituto de Biofísica Carlos Chagas Filho, Universidade Federal do Rio de Janeiro, Rio de Janeiro, Brazil; ^2^Instituto de Microbiologia Paulo de Góes, Universidade Federal do Rio de Janeiro, Rio de Janeiro, Brazil; ^3^Instituto Nacional para Pesquisa Translacional em Saúde e Ambiente na Região Amazônica, Conselho Nacional de Desenvolvimento Científico e Tecnológico, Rio de Janeiro, Brazil

**Keywords:** classically-activated macrophage, leishmaniasis, M1 and M2 macrophages, nitric oxide, ODF, OPGL, RANKL, TRANCE

## Abstract

Macrophages host *Leishmania major* infection, which causes cutaneous Leishmaniasis in humans. In the murine model, resistance to infection depends on the host immunity mediated by CD4 T-cell cytokines and macrophages. In association to other stimuli, the Th1 cytokine IFN-γ induces NO-mediated microbial killing by M1/classically-activated macrophages. By contrast, the Th2 cytokine IL-4 promotes M2/alternatively activated macrophages, which express arginase-1 and shelter infection. Other cytokines, such as RANKL, might also participate in the crosstalk between T cells and macrophages to restrict parasite infection. RANKL and its receptor RANK are known to play an essential role in bone remodeling, by inducing osteoclatogenesis. It has also been shown that RANKL stimulates antigen-presenting cells, such as DCs and macrophages, to enhance T cell responses. Here we investigated how RANKL directly modulates the effector macrophage phenotypes and immunity to *L. major* parasites. We found that inflammatory peritoneal macrophages from B6 mice express RANK and M2 features, such as CD301 (MGL) and CD206 (mannose receptor). Nonetheless, treatment with RANKL or IFN-γ induced macrophage differentiation into more mature F40/80^hi^ macrophages able to produce IL-12 and TNF-α. In parallel, macrophages treated with RANKL, IFN-γ, or RANKL along with IFN-γ progressively downregulated the expression of the M2 hallmarks MGL, arginase-1, and CCL17. Moreover, a synergism between IFN-γ and RANKL enhanced inducible NO synthase (iNOS) expression and NO production by macrophages. These results are consistent with the idea that RANKL helps IFN-γ to induce a M2-like to M1 phenotype shift. Accordingly, concomitant treatment with RANKL and IFN-γ promoted macrophage-mediated immunity to *L. major*, by inducing NO and ROS-dependent parasite killing. Furthermore, by cooperating with IFN-γ, endogenous RANKL engages CD4 T-cell help toward *L. major*-infected macrophages to upregulate M1 and Th1 cytokine responses. Therefore, RANKL, in combination with IFN-γ, is a potential local therapeutic tool to improve immune responses in Leishmaniasis, by skewing M2-like into effector M1 macrophages.

## Introduction

The Receptor Activator of Nuclear Factor-Kappa B (RANK), a TNF receptor family member, and its ligand RANKL (also known as TRANCE, ODF, and OPGL) are crucial for bone homeostasis, by promoting osteoclast differentiation and activation (1–7). RANK initiates multiple signaling pathways, including NF-κB activation, that lead to gene transcription, iNOS expression, and NO production by osteoclast and macrophages (8–11). Deficiencies in RANK or RANKL cause osteopetrosis owing to abnormal osteoclastogenesis and bone remodeling, but also affect the immune system and lymphoid organogenesis, precluding accurate studies of immunity to pathogens in RANK/RANKL-deficient mice (12–17).

RANKL-RANK axis was first described in the context of immune responses, where T-cell derived RANKL promotes dendritic cell/macrophage function to increase T-cell activation (1, 2, 10, 18–22). Activated T cells and Th1, but not Th2 cells express TRANCE/RANKL (18, 23) and treatment with RANKL stimulates DCs to secrete IL-12 (18, 19). In line with this, a DC therapy with RANKL-treated DCs primed Th1 responses to microbial antigens (20). Conversely, the blockade of RANKL precluded T-cell priming for Th1 responses both *in vitro* (23) and *in vivo* (19, 24). RANKL also enhanced APC features and secretion of inflammatory cytokines by bone marrow-derived macrophages (BMDMs), but failed to upregulate IL-12 or high levels of iNOS expression (22, 25). How RANKL modulates macrophage effector functions has not been fully explored.

*Leishmania* spp. infection causes several clinical forms ranging from localized lesions to disseminated Leishmaniasis, a major health problem in developing countries, by affecting humans and animals. In the experimental cutaneous Leishmaniasis, immunity to *Leishmania major* infection depends on the Th1/Th2 cytokines produced by CD4 T cells that shape macrophage phenotype, by inducing either classically/M1 or alternatively/M2 activated macrophages (26–32). In resistant mouse strains, Th1 cytokines, such as IFN-γ and TNF-α induce NO production and parasite killing by M1 macrophages. Otherwise, the Th2 cytokine IL-4 increases arginase-1 expression and parasite replication within M2 macrophages in susceptible mice (32–38). Despite efforts and advances, effective vaccination and immunotherapy are not available.

A previous study investigated whether RANKL plays a role as a costimulatory molecule on immune responses to *L. major* parasites (24). Whereas CD40L-deficient mice are resistant to infection, the blockade of RANKL-RANK interactions precluded IL-12 production by antigen presenting cells and shifted protective Th1 into Th2 responses (24). Nonetheless, it has not been elucidated how RANKL directly affects macrophage effector phenotypes, as well as their ability to fight parasite infection.

Here we investigated the role of RANKL-RANK axis on M1/M2 phenotypes and on macrophage-mediated immunity to *L. major*. We showed that, whereas peritoneal inflammatory cells express a M2-like phenotype, RANKL has direct effects on macrophages, by promoting the expression of the M1 cytokines TNF-α and IL-12 and by reducing M2-features. Moreover, RANKL synergizes with IFN-γ to increase iNOS expression, NO production, and the control of *Leishmania* parasites. Furthermore, endogenous RANKL and IFN-γ promote CD4 T-cell help to infected macrophages and upregulate both M1 and Th1 responses to parasite infection.

## Materials and Methods

### Mice and Parasites

C57BL/6 (B6) and BALB/c mice were obtained from the Oswaldo Cruz Foundation (FIOCRUZ, Rio de Janeiro, Brazil) and maintained in the animal facility at the Federal University of Rio de Janeiro (UFRJ). The animal study was reviewed and approved by the Ethics Committee for Use of Animals at the Federal University of Rio de Janeiro (UFRJ). All experiments were conducted as in the protocol 078/16 (CEUA-UFRJ). *L. major* LV39 (MRHO/Sv/59/P) parasites were isolated from popliteal lymph nodes of infected BALB/c mice and maintained up to 4 wk at 28°C in Schneider's medium (Sigma-Aldrich, USA), supplemented with 2% sterile human urine, 2 mM of L-glutamine, 10 μg/mL of gentamicin, and 10% fetal bovine serum (FBS, Gibco BRL, South America). For macrophage or mouse infection, *Leishmania* parasites were cultured until stationary phase at 28°C in Schneider's medium.

### Mouse Infection

B6 mice, aging 6–8 weeks, were injected i.p. with 3 ×10^6^ *L. major* parasites and peritoneal macrophages were collected after 24 h for T-cell/macrophage cocultures. As a source for T cells, B6 mice were infected s.c. (at hind footpads) with 3 ×10^6^ *L. major* parasites and the spleens were removed at 5 w.p.i.

### Inflammatory Macrophages

Inflammatory macrophages were obtained 4 days after the i.p. injection of 3% thioglycolate broth. Peritoneal resident macrophages or inflammatory (recruited) macrophages were collected by peritoneal lavage. Inflammatory macrophages were cultured in DMEM (Invitrogen Life Technologies), supplemented with 2 mM glutamine, 5 ×10^5^ M 2-ME, 10 μg/mL gentamicin, 1 mM sodium pyruvate, and 0.1 mM MEM non-essential amino acids (culture medium) plus 10% FBS. Cells were processed and analyzed prior or after culture by flow cytometry and functional assays. Cultures were treated with the following reagents: 0.2–0.5 ng/mL of recombinant IFN-γ (R&D Systems, EUA), 20 ng/mL of recombinant RANKL (R&D Systems, EUA), 200 ng/mL of LPS from *Salmonella enterica* serovar Typhimurium (Sigma), 10 μM of Bay 11-7082 (Santa Cruz Biotechnology, Dallas, USA) or DMSO (Sigma), 1 mM of N6-(1-imioetil) lysine (L-NIL) from Sigma, 100 μM of deferoxamine (DFO, Sigma) or N-acetyl-L-cysteine (NAC, Sigma). Cultures were maintained up to 3 days at 37°C with 7% of CO_2_.

### Macrophage Infection and Parasite Load

Inflammatory (B6) macrophages were cultured in triplicates at 1.5–3.5 ×10^5^ cells/well in 48-well vessels or at 4 ×10^5^ cells/well on glass coverslips inserted in 24-well plates during 1 h, and then washed. Adherent macrophages were infected for 24 h at a 10:1 parasites/macrophage ratio. Cultures were washed for removal of extracellular parasites and additional non-adherent cells. Infected macrophages were treated with the indicated reagents and maintained in culture medium plus 10% FBS at 37°C and 7% CO_2_ for 3 days. In cultures established in 48-well vessels, supernatants were replaced by Schneider's medium. Macrophages were further cultured for at least 4 days at 28°C in a BOD incubator (Cienlab) for determination of parasite load. Parasites released in culture supernatants were then counted in a Beckman Coulter (USA) within a range of 3–6 μm, for exclusion of murine cells. For intracellular amastigotes assessment, coverslips were stained with Panotic kit (Laborclin) and parasites were counted within macrophages. Results are expressed as numbers of parasites/100 macrophages.

### Flow Cytometry

Peritoneal resident macrophages and inflammatory macrophages were prepared for flow cytometry. For phenotype analyses, inflammatory (B6) macrophages were cultured in triplicates at 2.0–2.5 ×10^6^ cells/well in 24-well plates, treated with the indicated reagents and maintained at 37°C with 7% CO_2_ during 48 h. Cells were washed in FACS buffer (PBS 0.01% NaN3 plus 2% FBS) and incubated with anti-CD16/CD32 (eBioscience, San Diego, CA, USA) for Fc blocking. We stained cells with allophycocyanin or FITC-labeled anti-F4/80, (eBiosciences, Chicago, IL, USA), Alexa Fluor 488-labeled anti-CD301 (MGL) mAb (AbD Serotec, Kidlington, UK) or control rat IgG2a mAb (R&D Systems, Minneapolis, MN, USA) or with allophycocyanin-labeled anti-CD301 (MGL) mAb or control rat IgG2b mAb (Biolegend, San Diego, CA, USA); PE-labeled anti-RANK mAb (BioLegend), PE-labeled anti-206 (mannose receptor) (Biolegend) or control rat IgG2a mAb (eBioscience). For intracellular staining, we washed, permeabilized, fixed, and stained cells with PE-labeled anti-IL-12p35 or control murine IgG1 mAb (R&D Systems), PE-labeled anti-NOS2 (iNOS) or control rat IgG2a mAb (eBioscience); FITC-labeled anti-Arginase-1 or control sheep IgG mAb (R&D Systems). Cells were acquired with the CellQuest software on a FACSCalibur system (BD Biosciences, San Jose, CA, USA). For analysis, we used the FlowJo software (TreeStar, Ashland, OR, USA). F4/80^+^ cells were first gated and then evaluated for CD301^+^ or CD206^+^ cells, for IL-12p35^+^ and CD301^+^ subsets or for iNOS^+^ and Arginase-1^+^ subsets, based on the exclusion of background staining with isotype control mAbs.

### CD4 T-Cell Purification

Spleens from B6 mice infected at hind footpad with *L. major* were removed and depleted of red blood cells by Tris-buffered ammonium chloride treatment, followed by nylon wool filtration to obtain enriched T-cell suspensions. Purified CD4 T-cells were obtained by negative selection with a mAb mix, containing anti-CD8, anti-B220, anti-CD11b, and anti-panNK mAbs (BD Biosciences or Ebioscience), and anti-rat IgG magnetic beads (Dynal, Oslo, Norway). Cell suspensions were 85–88% CD4^+^ cells, as stained for detection of residual CD8 T cells or B cells with anti-CD4, anti-CD5, and anti-CD19 (BD Biosciences) mAbs.

### Cocultured Macrophages and CD4 T Cells

For cocultures, peritoneal macrophages from *L. major*-injected B6 mice were plated in triplicates at 5 ×10^5^ cells/well in 48-well vessels during 1 h and then washed. Adherent macrophages were infected for 7 h at a 10:1 parasites/macrophage ratio. Cultures were washed for removal of extracellular parasites and additional non-adherent (cells/well). Purified CD4 T-cells (5 ×10^5^/well) from infected mice were added to infected macrophages at a 1:1 ratio in 48-well vessels. Cocultures were then treated or not with 10 μg/mL of anti-mouse CD254 (RANKL, Biolegend) or anti-mouse IFN-γ (BD Bioscience) during 48 h. Supernatants were collected for cytokine assays.

### Cytokine Assays

Culture supernatants were used for detection of NO (as bellow) and cytokines (IL-12p70, TNF-α, CCL17) by ELISA assay. For that, we used pairs of specific mAbs (R&D Systems, eBioscience or PeproTech), one of which was labeled with biotin, and then developed with streptavidin-alkaline phosphatase (Invitrogen Life Technologies) and p-nitrophenyl phosphate (PNPP substrate, Thermo Scientific Pierce, Waltham, MA, USA), according to manufacturers. Alternatively, Avidin horseradish peroxidase (eBioscience) and 3,3′,5,5′-tetramethylbenzidine (TMB substrate, eBioscience) were used for detection.

### Nitric Oxide

Production of NO was determined indirectly by the quantification of nitrites. Culture supernatants were mixed with Griess reagent (1% sulfanilamide, 0.1% naphthylethylenediamine dihydrochloride, 2% H3PO4; Sigma) in a 1:1 ratio. A standard curve with known concentrations of sodium nitrite (NaNO_2_) was used and the results were expressed as nitrites (μM). The optical density was determined at 540 nm on a plate spectrophotometer (Versa Max, Molecular Devices).

### Statistics

Results are expressed as the mean of technical replicates per treatment and S.E.M in figures. For parasite load, data were transformed to log of parasites per mL for statistical analysis. Data were analyzed by unpaired Student's two-tailed *t*-test, by using the GraphPad Prism (v. 6.0). Otherwise, results were analyzed by one-way ANOVA, followed by the indicated post-tests. The numbers of independent repeat experiments and significant differences are indicated in the figure legends.

## Results

### RANK Expression and Inflammatory Macrophage Phenotype

**Figure 1:**
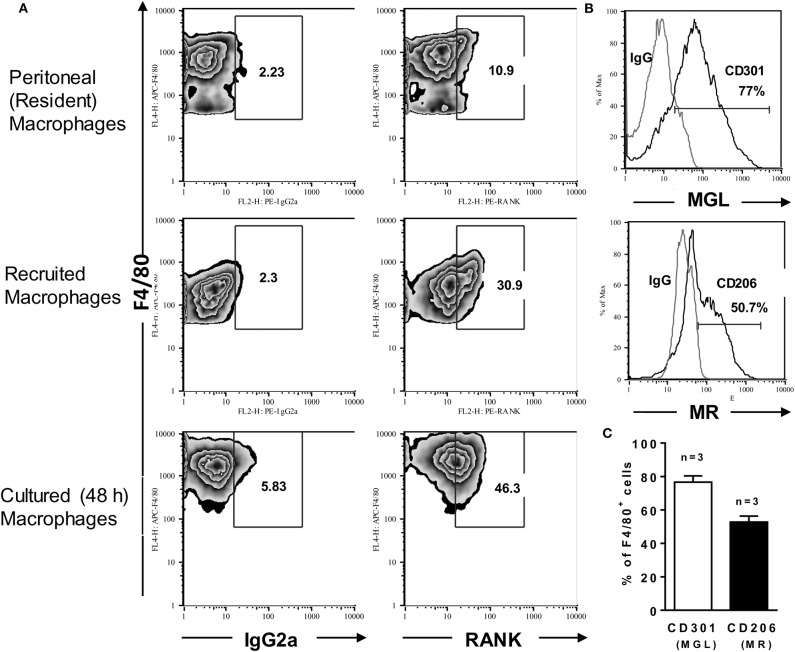
Inflammatory macrophages express RANK and a M2-like phenotype. Peritoneal (resident) macrophages and thioglycolate-elicited (inflammatory) macrophages from B6 mice were analyzed for the expression of RANK or for the M2 hallmarks CD301 (MGL) and CD206 (MR). **(A)** Peritoneal cells were stained with anti-F4/80 and anti-RANK or control IgG2a. Where indicated, inflammatory macrophages were cultured in medium only during 48 h before staining. **(B,C)** F4/80^+^ inflammatory macrophages were also stained with anti-CD301 or control IgG2b and anti-CD206 or control IgG2a, as indicated. In **(C)**, results are expressed as means and S.E.M. (*n* = 3 mice). Data are representative of 3 independent experiments.

To investigate the role of RANKL-RANK axis in macrophage-mediated immunity, we first determined RANK expression on F4/80^int^-inflammatory vs. F4/80^hi^-peritoneal (resident) macrophages from B6 mice (Figure 1A). After i.p. thioglycolate injection, about 20–30% of recruited (inflammatory) macrophages expressed RANK at higher levels compared with resident macrophages (Figure 1A). In unstimulated cultures, however, inflammatory macrophages upregulated both F4/80 and RANK expression (Figure 1A, lower panels). Next, we determined the effector phenotype of inflammatory (B6) macrophages (Figure 1 and Supplementary Figure 1). About 80% of elicited-peritoneal macrophages expressed the M2 marker MGL (CD301) and variable percentages of other M2 features (39), such as the mannose receptor (MR; CD206) (Figures 1B,C), the IL-4Rα subunit (CD124) (Supplementary Figure 1A), and arginase-1, but not the M1 hallmarks IL-12p35 (Supplementary Figure 1B) and iNOS (not shown). Moreover, a subset of CD206^hi^ macrophages expressed IL-10 (Supplementary Figure 1D). Thus, inflammatory peritoneal macrophages seem to express a M2-like phenotype.

### RANKL Upregulates F4/80 Expression by Macrophages

Since RANKL and IFN-γ have been used together to improve the expression of the costimulatory molecule CD86 in BMDMs (22), we first investigated whether RANKL and/or IFN-γ would also affect the expression of APC features (Supplementary Figure 2) in B6 inflammatory macrophages, which also expressed the MHC-class II I-A^b^ molecule (not shown). Here we observed that, compared with unstimulated macrophages, treatment with either RANKL and/or IFN-γ induced more mature macrophages, as assessed by higher F4/80 expression (Supplementary Figure 2, upper panels). We also found that untreated macrophages expressed the costimulatory CD80, CD86, and the coinhibitory PD-L1 molecules (Supplementary Figure 2A). Moreover, independent of treatment, macrophages remained positive for both costimulatory and coinhibitory molecules, although each treatment with RANKL and/or IFN-γ have differentially modulated the expression of CD80, CD86, and PD-L1 (Supplementary Figure 2B). It should be noticed, however, that macrophages treated with RANKL along with IFN-γ expressed the lowest levels of costimulatory molecules associated with the highest levels of the coinhibitory PDL-1 (Supplementary Figure 2B).

### RANKL Cooperates With IFN-γ to Induce Effector Macrophages

**Figure 2:**
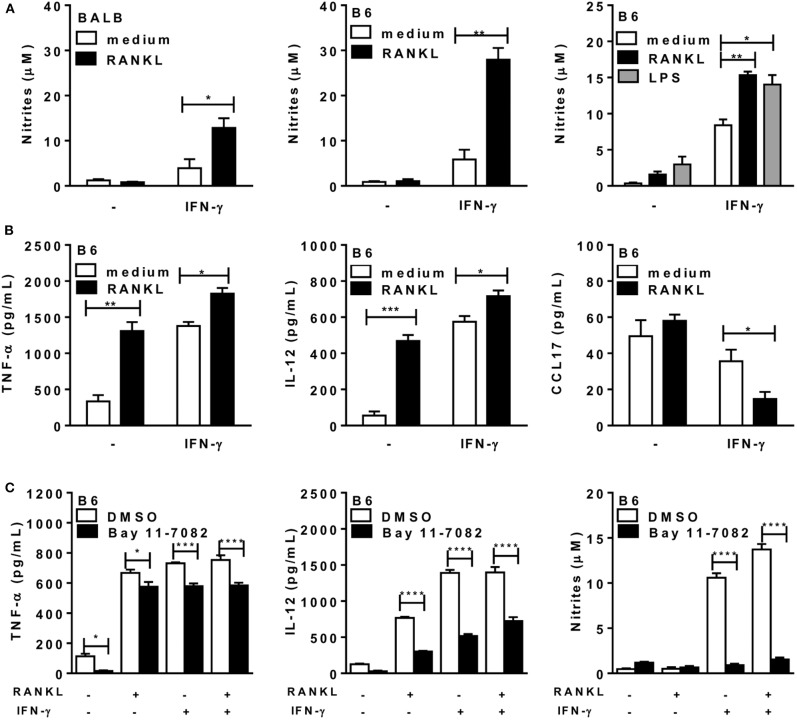
RANKL and IFN-γ increase cytokine and NO production by macrophages. Inflammatory macrophages from B6 or BALB/c mice were cultured in triplicates with medium or RANKL in the presence or absence of IFN-γ during 48 h. Some cultures also received the IKK-signaling inhibitor Bay 11-7082 or control diluent (DMSO). **(A,C)** Culture supernatants were tested for NO production by the Griess method. **(B,C)** Culture supernatants were assayed for TNF-α, IL-12p70, and CCL17 by ELISA. Results are expressed as means and S.E.M. For **(A,B)**, significant differences between macrophages treated with or without RANKL were analyzed by *t*-test. Data are representative of 3 independent experiments. In (**A**, right panel) LPS (200 ng/mL) was used as a positive control and the results were analyzed by one-way ANOVA, followed by Tukey post-test. In **(C)**, data were analyzed by one-way ANOVA, followed by Bonferroni post-test. Significant differences were indicated for *P <0.05, **P <0.01, ***P <0.001, and ****P <0.0001.

Next, we addressed the functional phenotypes of RANKL-treated macrophages. First, we observed that inflammatory macrophages from both *L. major-*susceptible BALB/c and resistant B6 mice expressed increased NO production only after treatment with both RANKL and IFN-γ (Figure 2A). The synergistic effect of suboptimal doses of IFN-γ (40) and RANKL (9) on NO production was comparable to treatment with LPS and IFN-γ (Figure 2A, right panel). In all the subsequent studies we employed only inflammatory macrophages from the B6 (Th1) mouse strain, which develops IFN-γ-dependent macrophage responses to *L. major* infection (41). We found that treatment with either RANKL or IFN-γ affected cytokine secretion by macrophages, by inducing TNF-α and IL-12p70, whereas RANKL reduced the M2 chemokine CCL17 only in the presence of IFN-γ (Figure 2B). Therefore, RANKL modulates macrophage function, either directly, by inducing the secretion of proinflammatory cytokines or in combination with IFN-γ to increase NO production and reduce a M2 chemokine. To address the role of NF-κB signaling in macrophage responses to RANKL, we used the IKK signaling inhibitor Bay 11-7082. Interestingly, IKK signaling inhibition only partially reduced TNF-α and IL-12 responses to different treatments, but completely blocked NO production in macrophages treated with IFN-γ or RANKL along with IFN-γ (Figure 2C). These results indicate that RANKL and/or IFN-γ differentially regulate gene transcription, as previously discussed (10, 38, 42), as well as that each M1-functional (IL-12, TNF-α, NO) response expresses variable degrees of NF-κB signaling dependence (Figure 2C).

### RANKL and IFN-γ Promote a M2-Like to M1 Phenotype Shift

**Figure 3:**
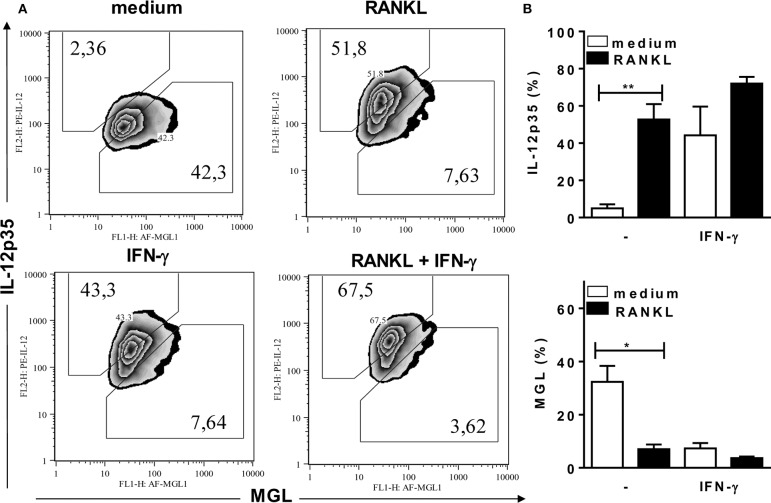
RANKL induces a M2-like to M1 phenotype shift. Inflammatory (B6) macrophages were cultured in triplicates with medium or RANKL in the presence or absence of IFN-γ. After 48 h, cells were harvested and stained with anti-F4/80, anti-CD301 (MGL), anti-IL-12p35, or control mAbs. **(A)** Plots depict F4/80^+^ cells as evaluated for CD301 (MGL) and intracellular IL-12p35 expression. **(B)** Results are expressed as means and S.E.M. Significant differences between macrophages treated with or without RANKL were analyzed by *t*-test and indicated for *P <0.05, and **P <0.01. Data are representative of 2 independent experiments.

**Figure 4:**
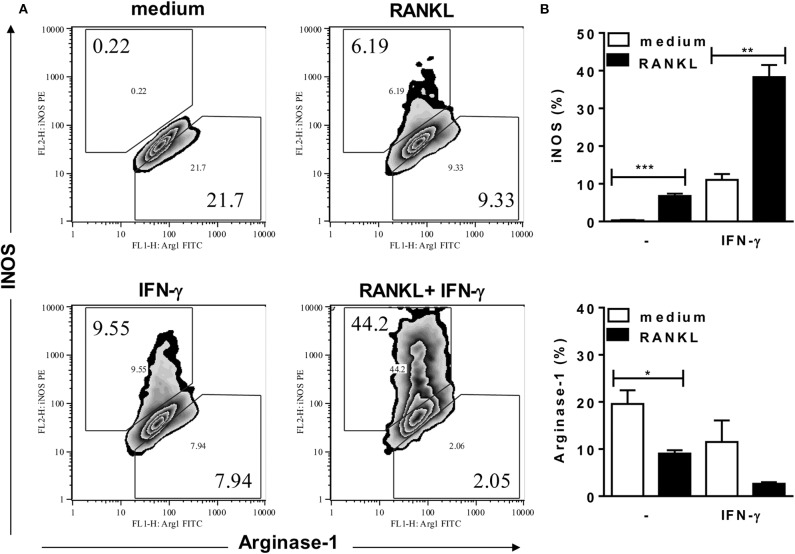
RANKL requires IFN-γ to induce high iNOS expression. Inflammatory (B6) macrophages were cultured in triplicates with medium or RANKL in the presence or absence of IFN-γ. After 48 h, cells were harvested and stained with anti-F4/80, anti-iNOS, anti-arginase-1 or control mAbs. **(A)** Plots depict F4/80^+^ cells as evaluated for intracellular iNOS and arginase-1 expression. **(B)** Results are expressed as means and S.E.M. Significant differences between macrophages treated with or without RANKL were analyzed by *t*-test and indicated for *P <0.05, **P <0.01, and ***P <0.001. Data are representative of 3 independent experiments.

To investigate the effects of RANKL on M1/M2 phenotypes of B6 inflammatory macrophages, we analyzed the expression of MGL (as a M2 marker) and intracellular IL-12p35 (as a M1 marker) (Figure 3), as well as the expression of iNOS and arginase-1 (Figure 4) enzymes, which control L-arginine metabolism (33). At steady state, unstimulated F4/80^+^ macrophages expressed a M2-like (MGL^+^) phenotype, but not IL-12p35 (Figures 3A,B). Conversely, treatment with RANKL reduced MGL and increased IL-12p35 expression (Figures 3A,B). More interesting, RANKL suppressed arginase-1 and cooperated with IFN-γ to increase iNOS expression at higher levels (Figures 4A,B). These findings might explain how RANKL and IFN-γ synergize to increase NO production by macrophages (Figure 2A). Next, we performed parallel cytometric analyses of multiple M1/M2 hallmarks, as well as secreted cytokines (Supplementary Figures 3, 4). In unstimulated cultures, at least 80% of macrophages expressed MGL (but not iNOS) (Supplementary Figure 4A) and different percentages of the M2 markers CD206, CD124, and arginase-1 (Supplementary Figure 3). The M2 hallmarks and the secretion of CCL17 chemokine were progressively downregulated by RANKL, IFN-γ, and RANKL along with IFN-γ (Supplementary Figures 3, 4). By contrast, iNOS expression and IL-12 production were upregulated in treated macrophages (Supplementary Figures 4A,C), indicating a M2-like to M1 shift. Moreover, we observed a subset of double positive macrophages, which expressed both MGL and iNOS in cultures treated with RANKL (Supplementary Figure 4B). Therefore, in the presence of RANKL, M2-like macrophages can give rise to M1-intermediate cells (with lower iNOS expression), whereas cooperation with IFN-γ is required to induce a bona fide M1 phenotype with higher iNOS expression and reduced M2 features.

### RANKL and IFN-γ Help Macrophages to Control Infection

**Figure 5:**
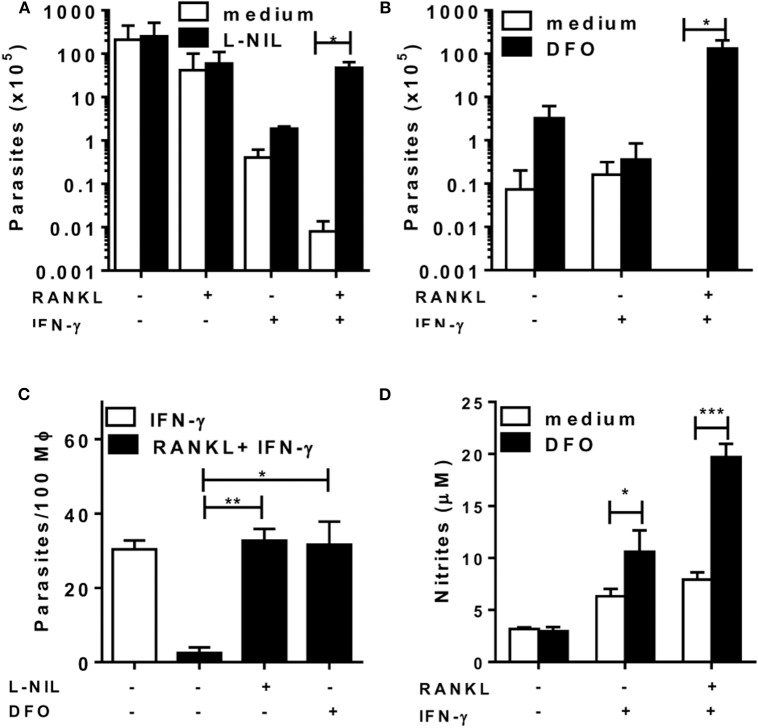
RANKL and IFN-γ induce *L. major*-parasite control. Inflammatory (B6) macrophages were infected with *L. major* (1:10) during 24 h and then treated with RANKL, IFN-γ, L-NIL (1 mM) or DFO (100 μM) during 72 h. **(A–C)** Parasitic burden was determined as **(A,B)** released promastigotes (parasites/mL) or **(C)** by counting amastigotes within macrophages. **(D)** Culture supernatants were tested for NO production by the Griess method. Results are expressed as means and S.E.M. Significant differences between macrophages treated without and with L-NIL or DFO were analyzed by *t*-test and indicated for *P <0.05, **P <0.01, and ***P <0.001. Data are representative of 2 independent experiments.

Next, we infected macrophages with *L. major* to test whether RANKL could affect macrophage-mediated immunity for parasite control (Figure 5). Although treatment with RANKL alone was not able to reduce parasite infection, it did enhance *L. major* control by IFN-γ-treated macrophages (Figure 5). Moreover, iNOS inhibition completely blocked parasite killing by macrophages treated with RANKL plus IFN-γ (Figures 5A,C). Nonetheless, the use of ROS inhibitors DFO (Figures 5B,C) or NAC (not shown) also blocked the control of *L. major* parasites, although NO production has actually increased in these conditions (Figure 5D). These results indicate that, although necessary, NO was not sufficient to control parasite infection by effector M1 macrophages generated in the presence of RANKL and IFN-γ.

### RANKL and IFN-γ Cooperate in CD4 T-Cell Help to Macrophages

**Figure 6:**
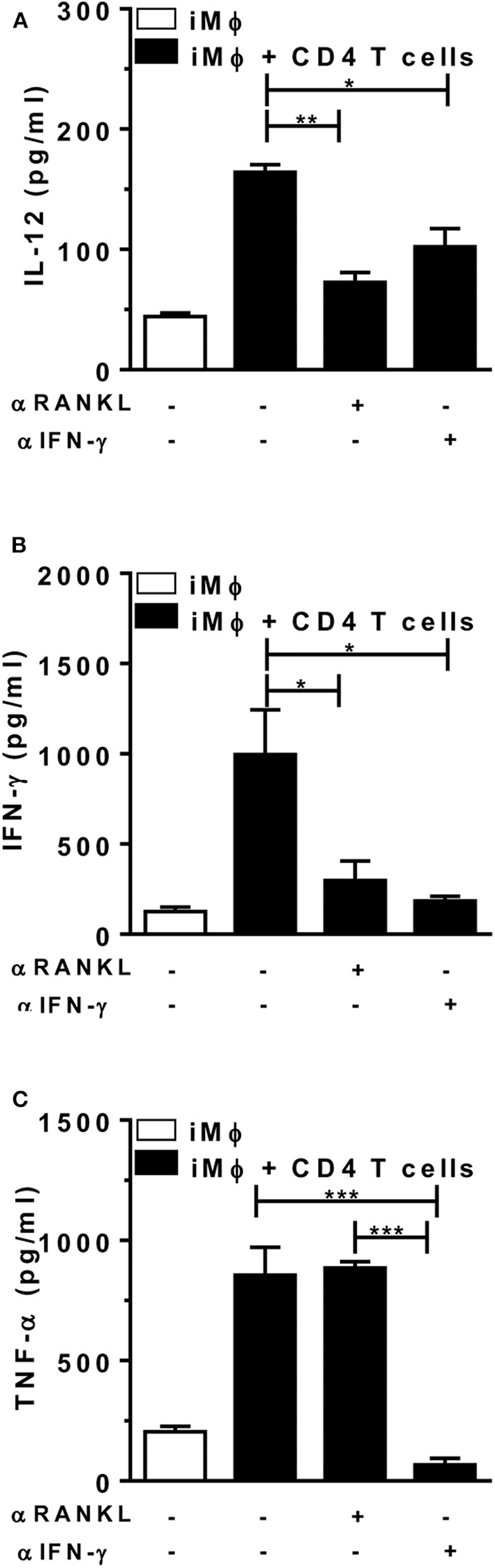
Endogenous RANKL is involved in CD4 T-cell help to macrophages. Peritoneal (B6) macrophages (from mice injected i.p. with *L. major*) were infected *in vitro* and then cultured alone or with CD4 T cells from *L. major*-infected mice (at 5 w.p.i.). Cultures were treated or not with anti-RANKL or anti-IFN-γ-neutralizing antibodies. **(A-C)** After 48 h, culture supernatants were collected and assayed for **(A)** IL-12p70, **(B)** IFN-γ, and **(C)** TNF-α by ELISA. Results are expressed as means and S.E.M. Results from cocultures were analyzed by one-way ANOVA, followed by Tukey post-test. Significant differences were indicated for *P <0.05, **P <0.01, ***P <0.001.

Finally, we addressed the role of paracrine/surface RANKL and IFN-γ in the crosstalk between T cells and macrophages at the effector phase of immune responses, by using neutralizing mAbs against RANKL and IFN-γ. For that, we purified CD4 T-cells from *L. major*-infected mice, which were cocultured with peritoneal macrophages from mice injected with *L. major*. Macrophages were infected *in vitro* in order to present antigens to CD4 T cells and upregulate IL-12, IFN-γ, and TNF-α responses (Figure 6). Treatment with anti-RANKL reduced both IL-12 and IFN-γ, but not TNF-α in cocultures, whereas anti-IFN-γ reduced IL-12 secretion and completely blocked the production of TNF-α (Figures 6A–C). These results are consistent with the idea that endogenous (T-cell derived) RANKL and IFN-γ might help macrophages to produce IL-12 and upregulate Th1 responses.

## Discussion

RANKL might play a costimulatory role in the immune responses mediated by APCs and T cells during *L. major* infection, as previously suggested (24). Now, we expand this view, by proposing that RANKL works in concert with IFN-γ to improve macrophage effector mechanisms for macrophage-mediated immunity and inflammatory responses. Whereas, inflammatory macrophages from B6 mice express RANK and a M2-like phenotype, RANKL induces their differentiation into F4/80^hi^-mature macrophages, able to secrete inflammatory cytokines. Moreover, RANKL cooperates with IFN-γ to skew M2-like into M1 macrophages, which express iNOS, NO production, and parasite killing, as discussed below.

RANKL-RANK axis is known to play a major role in osteoclastogenesis. In combination with other cytokines, RANKL promotes osteoclast maturation, but it also induces regulatory mechanisms, such as NO production by osteoclasts to maintain bone homeostasis (9). In certain conditions, RANKL might also induce iNOS expression and NO production by macrophages (9, 11). Moreover, the presence of RANKL in the vicinity of iNOS-expressing macrophages in rat bones indicates that, in addition to the role in osteoclastogenesis, RANKL might promote osteogenesis, by inducing M1 macrophages (25). Conversely, depending on the tissue environment and cell origin, RANKL might induce M2 macrophages (43), as well as either IL-12- or IL-10-producing DCs (44), and play tolerogenic/immunoregulatory effects *in vivo*. These examples highlight how RANKL affects different immune responses in a context/cell-dependent fashion, and that specific features of each model/disease should be considered to design studies to address the role of RANKL on immune cells.

It has been reported that RANKL has direct effects on DCs, by inducing IL-12 secretion and APC function, both relevant for T-cell activation and vaccine development (18, 20). Similarly, whereas B6 inflammatory macrophages expressed a M2-like phenotype, we found that RANKL directly induced IL-12 and TNF-α responses. Moreover, we show, for the first time, that RANKL favors M1 phenotype, by inducing IL-12, but suppressing MGL, arginase-1, and other M2 features. By contrast, although RANKL (at suboptimal doses) may have a limited effect on iNOS expression (22, 25) and NO production, it does act in synergism with IFN-γ to induce iNOS/NO by macrophages. It is reasonable to propose that a higher threshold for potential harmful NO responses demands either multiple signaling pathways initiated by both RANKL and IFN-γ, including NF-κB activation [our data and Guerrini and Takayanagi (10)], as well as epigenetic and post-transcriptional mechanisms induced by IFN-γ to upregulate iNOS expression (38).

Here we show that RANKL and IFN-γ promote the differentiation of M1 macrophages, which might be able to activate IFN-γ-producing Th1 cells to further upregulate M1 effector mechanisms. In contrast to Park et al. (22), we found that treatment with RANKL and IFN-γ upregulated the coinhibitory PD-L1 molecule and reduced macrophage expression of costimulatory features required for T-cell priming. It is likely that the differentiation into effector M1 macrophages is followed by reduced ability to prime naïve T cells along with increased regulatory activity on effector PD-1^+^ T cells, potentially deleterious to protect against *Leishmania* infection (45). In order to understand the role of RANKL and IFN-γ at the effector phase of Th1 and M1 responses, we investigated the crosstalk between CD4 T cells and macrophages from *L. major*-infected mice, by using anti-RANKL and anti-IFN-γ to neutralize endogenously produced cytokines. Whereas, both RANKL and IFN-γ concurred for IL-12 responses, RANKL was also required for IFN-γ production by T cells. Here, we highlighted a previously unexplored role for RANKL and IFN-γ in the interplay between Th1 cells and infected macrophages, by upregulating each other cytokine responses to parasite infection. Altogether, these results are relevant for both T-cell and macrophage-mediated immunity to *L. major* infection and expand previous findings on the role of RANKL as a costimulatory molecule that stimulate APC-T cell crosstalk *in vivo* (24). In a model, where CD40L-deficient mice were resistant to *L. major*, the blockade of RANKL induced susceptibility to infection, by reducing Th1 responses (24). Based on our findings, we suggest that in addition to possible defective APC/T-cell responses, mice lacking RANKL/RANK axis might have limited M1 effector mechanisms for parasite killing, such as iNOS expression and NO production.

We further addressed the role of RANKL on the potential effector mechanisms for macrophage-mediated immunity toward *L. major* parasites. We show that RANKL and IFN-γ cooperate to help parasite killing by B6 inflammatory macrophages in a NO-dependent fashion. More interesting, whereas NO plays a role as an effector molecule, ROS-related mechanisms might also concur for parasite killing by macrophages stimulated with RANKL and IFN-γ. Accordingly, *L. major* infection is able to induce ROS production by macrophages [data not shown and Goncalves et al. (46)]. Moreover, inhibition of peroxidase, by using DFO (or NAC), suppressed parasite killing even in the presence of increased NO production. These findings are consistent with the idea that both NO and ROS are necessary to kill *L. major*, through a peroxinitrite-dependent mechanism (47). Nonetheless, in contrast to these direct effects of NAC/DFO on infected B6 macrophages stimulated with RANKL and IFN-γ, the use of NAC to treat susceptible BALB/c mice reduced both lesions and parasite burden, most likely by increasing Th1 and M1-cytokine responses to *L. major* infection (48).

It has been reported that RANKL can act as an adjuvant in vaccines, by enhancing T-cell activation and Th1 responses induced by DCs (20, 49). Interestingly, a DNA vaccine containing RANKL gene stimulated antigen-specific T-cell response to *Trypanosoma cruzi* infection (49). Here we show that RANKL was able to act on inflammatory immature cells to induce more mature macrophages that express F4/80 at higher level. Moreover, RANKL costimulated the effector arm of Th1 responses, by potentiating the M1 effector mechanisms and parasite killing by infected macrophages. It is worth investigating whether RANKL could counteract a maturation deficit, which underlies defective macrophage immunity to *Leishmania* infection in susceptible mice (50–53). According to this idea, we found that RANKL and IFN-γ also enhance NO production in susceptible BALB/c macrophages. Hence, RANKL, in combination with IFN-γ, has potential use for local therapy, as previously suggested for IL-12 (54), as well as for therapeutic vaccines, reducing the risk of systemically side effects on bone homeostasis. Altogether, our results indicate that RANKL produced by activated T cells might costimulate T-cell-macrophage crosstalk: 1- to upregulate IL-12 and induction of Th1 responses; 2- to promote M1-mediated immunity in concert with IFN-γ. Therefore, RANKL might help protective Th1-mediated immune responses to *Leishmania* infection, by directly acting on macrophages to shift M2-like into effector M1 macrophages.

## Data Availability Statement

All datasets generated for this study are included in the article/Supplementary Material.

## Ethics Statement

The animal study was reviewed and approved by the Ethics Committee for Use of Animals at the Federal University of Rio de Janeiro (UFRJ).

## Author Contributions

TR performed and designed experiments, analyzed data, and co-wrote the manuscript. NV, MC-P, LF-C, and AF performed cell culture, cytokine, and flow cytometry assays. UL analyzed and discussed data. GD designed the research, supervised the experiments, and analyzed data. ML designed the research, supervised the experiments, analyzed data, and wrote the manuscript. TR, NV, MC-P, LF-C, UL, and AF approved the final version of the manuscript.

## Conflict of Interest

The authors declare that the research was conducted in the absence of any commercial or financial relationships that could be construed as a potential conflict of interest.
