# Supplementary material for: RANK Ligand Helps Immunity to Leishmania major by Skewing M2-Like Into M1 Macrophages
Source: Front Immunol. 2020 May 12;11:886. doi: 10.3389/fimmu.2020.00886 (PMC7235166; doi:10.3389/fimmu.2020.00886)
Supplement: Supplementary file 1 [file Data_Sheet_1.pdf]

Supplementary Material

1 Supplementary Data

Antibodies used for flow cytometry

For surface staining we used, allophycocyanin- or FITC-labeled F4/80; allophycocyanin-labeled anti-CD301 (MGL) and IgG2b mAbs; allophycocyanin-labeled anti-PDL-1 (Biolegend, San Diego, CA, USA) and anti-CD86 (eBioscience, San Diego, CA, USA) mAbs; PE-labeled anti-CD80 (eBioscience) and control hamster IgG (BD Biosciences, San Jose, CA, USA) mAbs; PE-labeled anti-CD206 (Biolegend) and anti-CD124 (BD Biosciences) mAbs. For intracellular staining, we employed the following mAbs: PE-labeled anti-IL-12p35 and control murine IgG1 mAbs; PE-labeled anti-NOS2 (iNOS) and control rat IgG2a; FITC-labeled anti-arginase-1 and control sheep IgG mAbs; allophycocyanin-labeled anti-IL-10 (BD Biosciences) mAb.

2 Supplementary Figures and tables

Supplementary Figure 1 - Inflammatory macrophages express a M2-like phenotype. Inflammatory macrophages from B6 mice were gated in F4/80$^+$ cells and analyzed for the expression of CD124 (IL-4Rα subunit), arginase-1, IL-12p35, CD206 (MR), and the respective control mAbs. (A) Histograms correspond to CD124$^+$ macrophages and control IgG2a staining. (B) Plots depict F4/80$^+$ cells as evaluated for the expression of intracellular arginase-1 and IL-12p35, as well as staining with the control mAbs. (C) Histograms correspond to CD206$^+$ macrophages and control IgG2a staining, where CD206^{high} and CD206^{low} subsets were defined. (D) Plots depict CD206^{high} and CD206^{low} macrophage subsets, which were analyzed for CD206 and intracellular IL-10 expression.

Supplementary Figure 2 - Inflammatory macrophages express both costimulatory and coinhibitory molecules. Inflammatory (B6) macrophages were cultured in triplicates with medium or RANKL in the presence or absence of IFN-γ. After 48 h, cells were harvested and stained with anti-F4/80, anti-CD80 (B7-1), anti-CD86 (B7-2) or anti-PDL-1. (A) F4/80$^+$ macrophages from unstimulated or RANKL-treated (upper panel only) cultures were analyzed for the expression of F4/80, CD80, CD86, and PDL-1. Staining with hIgG was used for the control of CD80 expression, otherwise, histograms represent unstained cells used for the control of CD86 and PDL-1 expression. (B) F4/80$^+$ cells were gated and evaluated for the expression of F4/80, CD80, CD86, and PDL-1, as mean (median) of fluorescence intensity (MFI). Results are expressed as means and S.E.M. Data were analyzed by one-way ANOVA followed by Dunnett post-test. Significant differences between unstimulated cultures and those treated with RANKL and/or IFN-γ were indicated for $P<0.01$ (**), $P<0.001$ (***), and $P<0.0001$ (****).

Supplementary Figure 3 - RANKL and IFN-γ downregulate the M2-like phenotype. Inflammatory (B6) macrophages were cultured in triplicates with medium or RANKL in the presence or absence of IFN-γ. After 48 h, cells were harvested and stained with anti-F4/80, anti-CD206 (MR), anti-CD124 (IL-4Rα subunit), and anti-arginase-1, as well as the respective control mAbs. Plots depict F4/80$^+$

cells as evaluated for the expression of surface CD206 and CD124, as well as intracellular arginase-1. Staining with IgG2a was used for the control of CD206 and CD124 expression, whereas sIgG controlled intracellular arginase-1 expression.

Supplementary Figure 4 - RANKL and IFN-γ skew M2-like into M1 macrophages. Inflammatory (B6) macrophages were cultured in triplicates with medium or RANKL in the presence or absence of IFN-γ during 48 h. (A, B) Cells were harvested and stained with anti-F4/80, anti-CD301 (MGL), iNOS or control IgG mAbs. Plots depict F4/80$^+$ cells as evaluated for the expression of surface CD301 and/or intracellular iNOS. (C) Culture supernatants were assayed for IL-12p70 and CCL17 by ELISA. Results are expressed as means and S.E.M. Data were analyzed by one-way ANOVA followed by Dunnett post-test. Significant differences between unstimulated and treated cultures were indicated for P<0.05 (*), P<0.01 (**), and P<0.001 (***).

2.1. Supplementary Figures

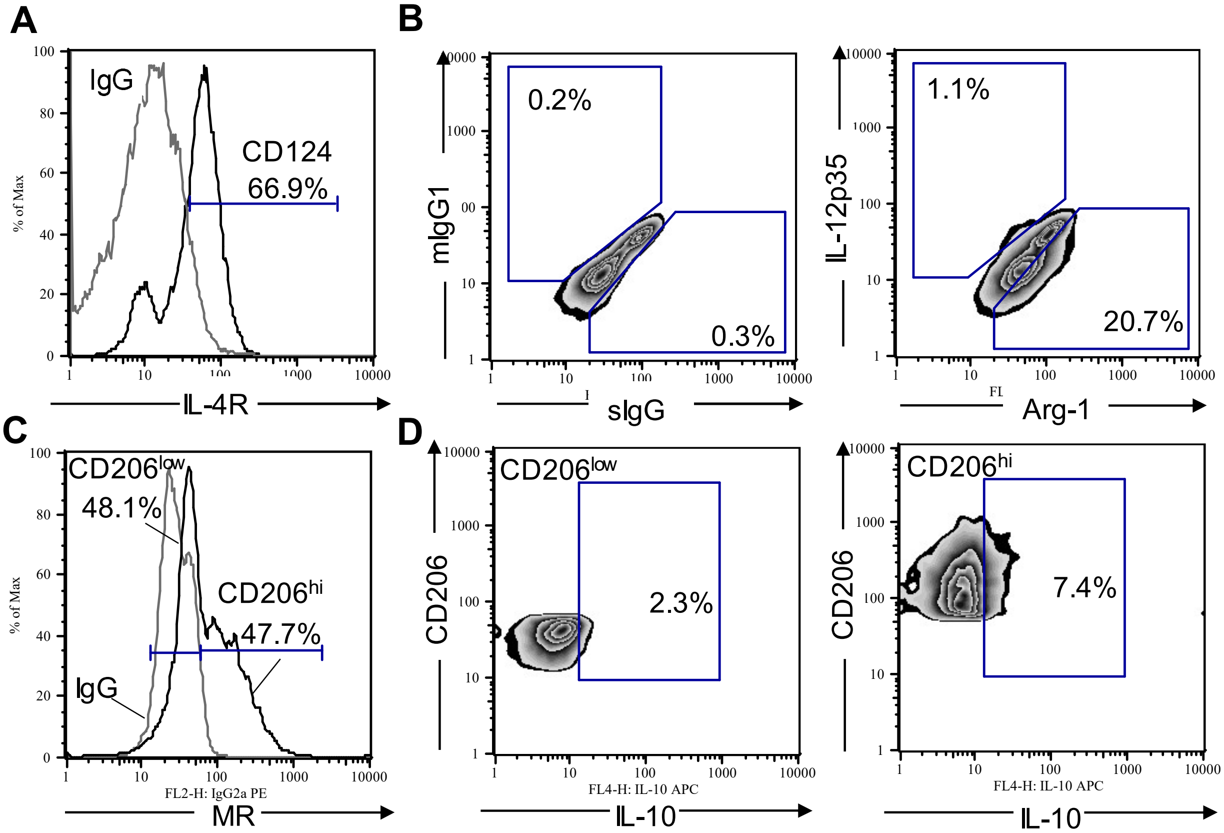

Supplementary Figure 1 - Inflammatory macrophages express a M2-like phenotype. Inflammatory macrophages from B6 mice were gated in F4/80$^+$ cells and analyzed for the expression of CD124 (IL-4Rα subunit), arginase-1, IL-12p35, CD206 (MR), and the respective control mAbs. (A) Histograms correspond to CD124$^+$ macrophages and control IgG2a staining. (B) Plots depict F4/80$^+$ cells as evaluated for the expression of intracellular arginase-1 and IL-12p35, as well as staining with the control mAbs. (C) Histograms correspond to CD206$^+$ macrophages and control IgG2a staining, where CD206^{high} and CD206^{low} subsets were defined. (D) Plots depict CD206^{high} and CD206^{low} macrophage subsets, which were analyzed for CD206 and intracellular IL-10 expression.

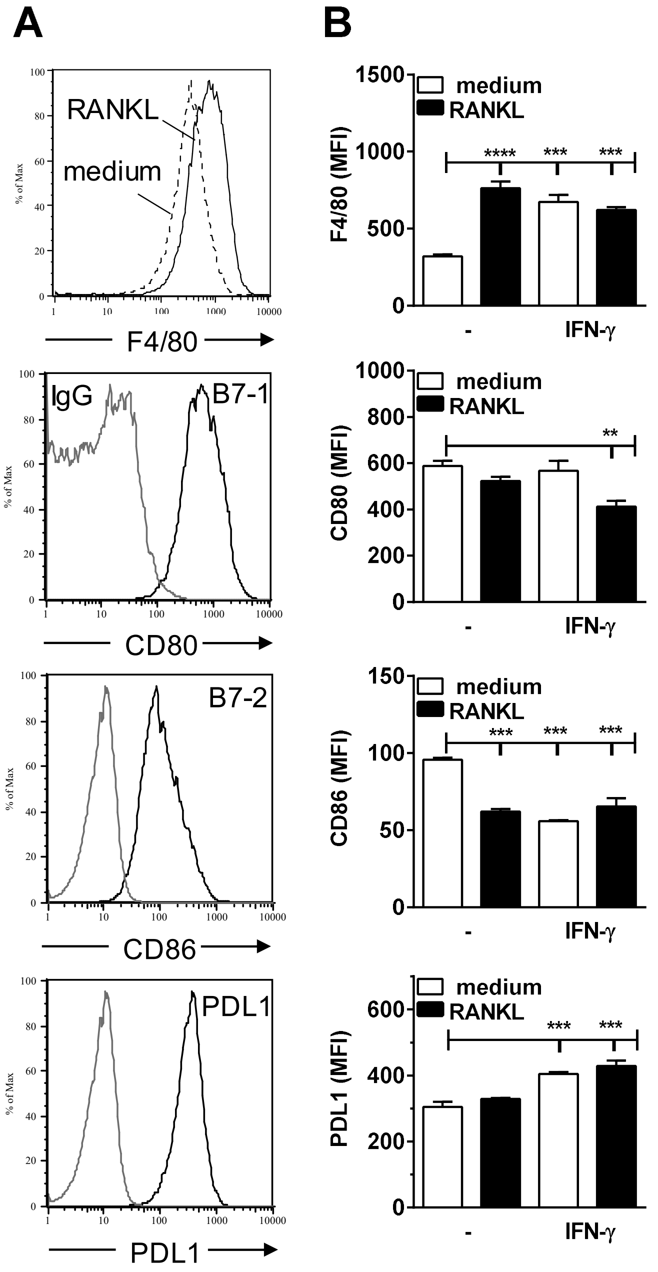

Supplementary Figure 2 - Inflammatory macrophages express both costimulatory and coinhibitory molecules. Inflammatory (B6) macrophages were cultured in triplicates with medium or RANKL in the presence or absence of IFN-γ. After 48 h, cells were harvested and stained with anti-F4/80, anti-CD80 (B7-1), anti-CD86 (B7-2) or anti-PDL-1. (A) $F4/80^+$ macrophages from unstimulated or RANKL-treated (upper panel only) cultures were analyzed for the expression of F4/80, CD80, CD86, and PDL-1. Staining with hIgG was used for the control of CD80 expression, otherwise, histograms represent unstained cells used for the control of CD86 and PDL-1 expression. (B) $F4/80^+$ cells were gated and evaluated for the expression of F4/80, CD80, CD86, and PDL-1, as mean (median) of fluorescence intensity (MFI). Results are expressed as means and S.E.M. Data were analyzed by one-way ANOVA followed by Dunnett post-test. Significant differences between unstimulated cultures

and those treated with RANKL and/or IFN-γ were indicated for P<0.01 (**), P<0.001 (***), and P<0.0001 (****).

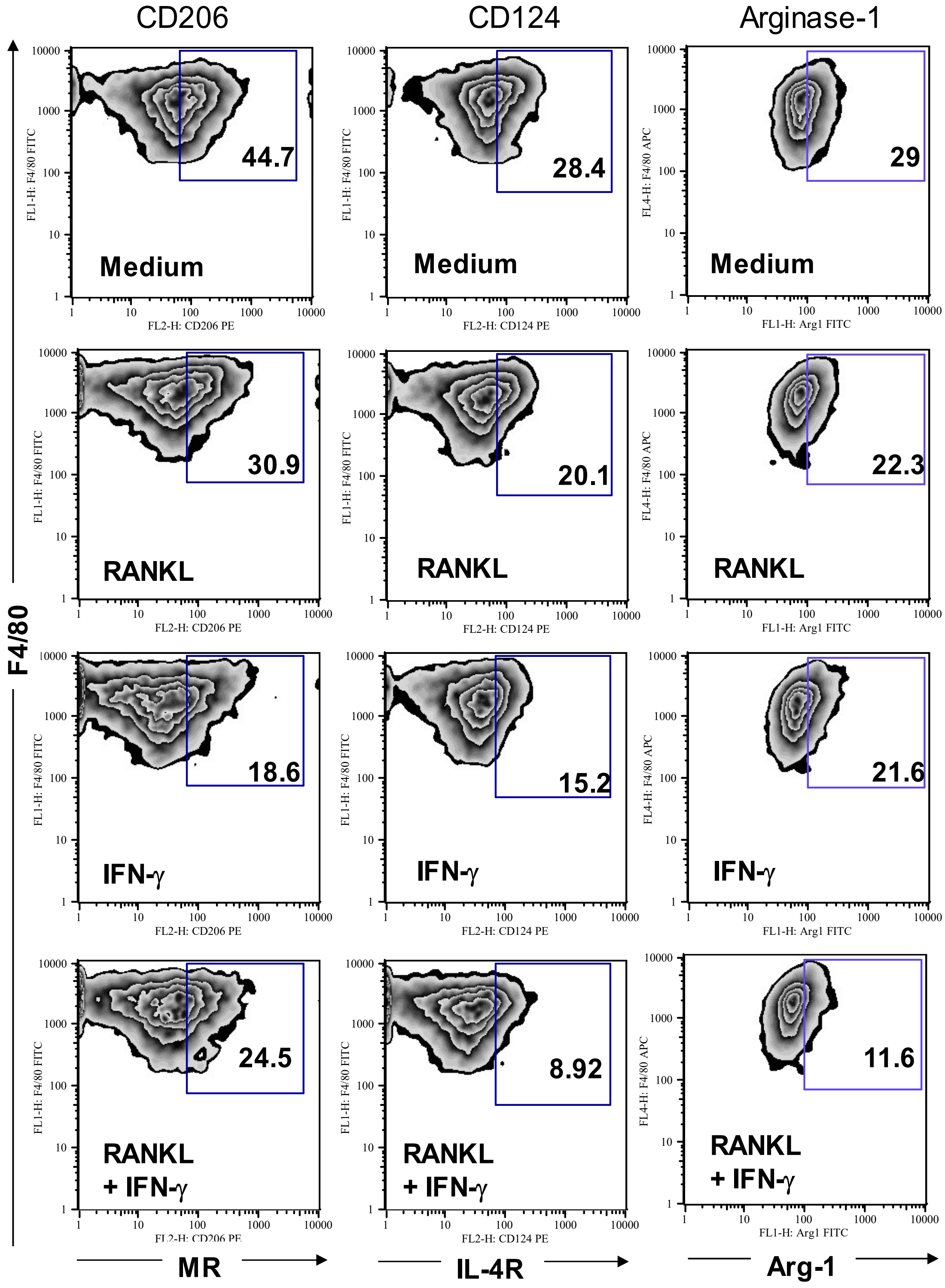

Supplementary Figure 3 - RANKL and IFN-γ downregulate the M2-like phenotype. Inflammatory (B6) macrophages were cultured in triplicates with medium or RANKL in the presence or absence of IFN-γ. After 48 h, cells were harvested and stained with anti-F4/80, anti-CD206 (MR), anti-CD124 (IL-4Rα subunit), and anti-arginase-1, as well as the respective control mAbs. Plots depict F4/80$^+$ cells as evaluated for the expression of surface CD206 and CD124, as well as intracellular arginase-1. Staining with IgG2a was used for the control of CD206 and CD124 expression, whereas sIgG controlled intracellular arginase-1 expression.

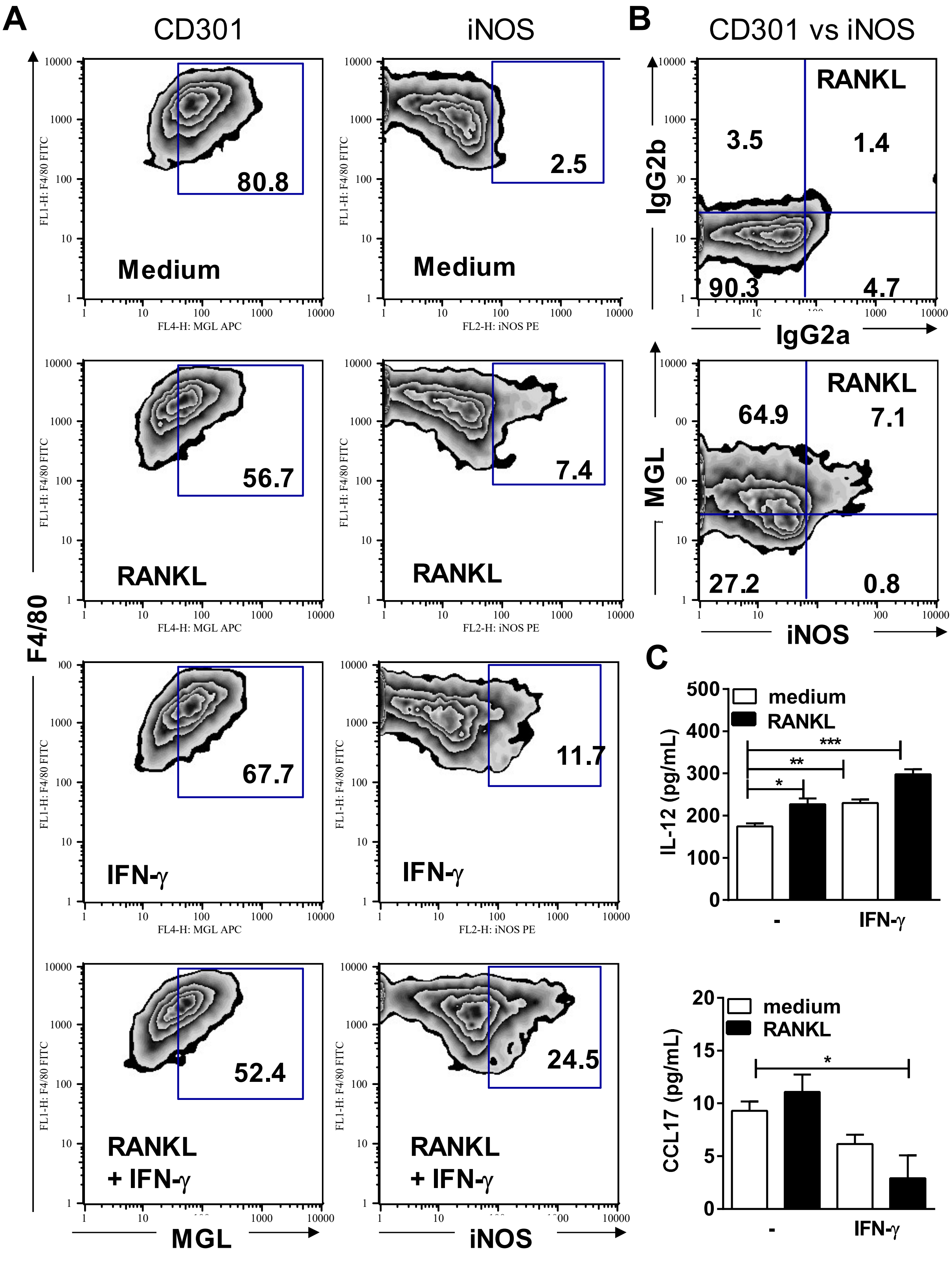
A
CD301
iNOS
B
CD301 vs iNOS
F4/80
MGL
iNOS
Medium
80.8
2.5
RANKL
56.7
7.4
IFN-γ
67.7
11.7
RANKL
+ IFN-γ
52.4
24.5
FL1-H: F4/80 FITC
FL4-H: MGL APC
FL2-H: iNOS PE
IgG2b
IgG2a
3.5
1.4
90.3
4.7
64.9
7.1
27.2
0.8
C
medium
RANKL
IL-12 (pg/mL)
CCL17 (pg/mL)
-
IFN-γ
*
**
***

Supplementary Figure 4 - RANKL and IFN-γ skew M2-like into M1 macrophages. Inflammatory (B6) macrophages were cultured in triplicates with medium or RANKL in the presence or absence of IFN-γ during 48 h. (A, B) Cells were harvested and stained with anti-F4/80, anti-CD301 (MGL), iNOS or control IgG mAbs. Plots depict F4/80$^+$ cells as evaluated for the expression of surface CD301 and/or intracellular iNOS. (C) Culture supernatants were assayed for IL-12p70 and CCL17 by ELISA. Results are expressed as means and S.E.M. Data were analyzed by one-way ANOVA followed by Dunnett post-test. Significant differences between unstimulated and treated cultures were indicated for $P<0.05$ (*), $P<0.01$ (**), and $P<0.001$ (***).
